# Ten simple rules to improve academic work–life balance

**DOI:** 10.1371/journal.pcbi.1009124

**Published:** 2021-07-15

**Authors:** Michael John Bartlett, Feyza Nur Arslan, Adriana Bankston, Sarvenaz Sarabipour

**Affiliations:** 1 Scion, Rotorua, New Zealand; 2 Institute of Science and Technology Austria, Klosterneuburg, Austria; 3 Future of Research, Pittsfield, Massachusetts, United States of America; 4 Institute for Computational Medicine and Department of Biomedical Engineering, Johns Hopkins University, Baltimore, Maryland, United States of America; Dassault Systemes BIOVIA, UNITED STATES

## Introduction

The ability to strike a perceived sense of balance between work and life represents a challenge for many in academic and research sectors around the world. Before major shifts in the nature of academic work occurred, academia was historically seen as a rewarding and comparatively low-stress working environment [1]. Academics today need to manage many tasks during a workweek. The current academic working environment often prioritizes productivity over well-being, with researchers working long days, on weekends, on and off campus, and largely alone, potentially on tasks that may not be impactful. Academics report less time for research due to increasing administrative burden and teaching loads [1–3]. This is further strained by competition for job and funding opportunities [4,5], leading to many researchers spending significant time on applications, which takes away time from other duties such as performing research and mentorship [1,2]. The current hypercompetitive culture is particularly impactful on early career researchers (ECRs) employed on short-term contracts and is a major driver behind the unsustainable working hours reported in research labs around the world, increases in burnout, and decline in satisfaction with work–life balance [6–10]. ECRs may also find themselves constrained by the culture and management style of their laboratory and principal investigator (PI) [11–12]. Work–life balance can be defined as an individual’s appraisal of how well they manage work- and nonwork-related obligations in ways that the individual is satisfied with both, while simultaneously maintaining their health and well-being [13]. Increasing hours at work can conflict with obligations outside of work, including but not limited to family care commitments, time with friends, time for self-care, and volunteering and community work. The increasing prevalence of technology that allows work to be out of the office can also exacerbate this conflict [14,15].

The academic system’s focus on publications and securing grant funding and academic positions instead of training, mentoring, and mental health has skewed the system negatively against prioritizing “The whole scientist” [5,16]. Research focused on the higher education sector has revealed that poor work–life balance can result in lower productivity and impact, stifled academic entrepreneurship, lower career satisfaction and success, lower organizational commitment, intention to leave academia, greater levels of burnout, fatigue and decreased social interactions, and poor physical and mental health, which has become increasingly prevalent among graduate students [1,17–22]. For instance, a recent international survey of over 2,000 university staff views on work–life balance found that many academics feel stressed and underpaid and struggle to fit in time for personal relationships and family around their ever-growing workloads [20]. These systemic issues are making it increasingly difficult to maintain an efficient, productive, and healthy research enterprise [23].

In the academic context, work–life balance needs to be examined with regard to spatial and temporal flexibility, employment practices, and employee habits. The need to improve work–life balance is recognized for researchers at all career stages [7,22,24,25]. While there is a growing literature providing specific strategies to cope with busy academic life [26–28], collating these disparate advice pieces into a coherent framework is a daunting task and few capture multifaceted advice by ECRs for ECRs. Departments and institutes need to contribute to improving research practices for academics at all levels on the career ladder [29,30]. PIs and mentors can promote healthier environments in their laboratories by respecting boundaries and providing individuals with greater autonomy over their own working schedule [11,12,31–33]. However, institutions do not typically prioritize work–life balance, leading to the loss of valuable talent in the research pipeline. The power dynamics within academia are evident now more than ever, with ECRs lacking agency at multiple time points and in controlling many aspects of their training. This may be especially true for trainees from underrepresented backgrounds, who face additional hurdles to their professional advancement in the current academic environment while attempting to maintain work–life balance. Furthermore, academia, in general, does not always value the aspects of a researcher’s job that the researcher finds important such as teaching, mentoring, and service. Thus, the experience of individual researchers regarding work–life balance will vary depending on multiple factors [34–39], including personal circumstances and satisfaction with aspects of life outside of work [40]. It is therefore unlikely that there is a “one size fits all” approach to effectively address work–life balance issues.

In order to support ECRs in maintaining work–life balance, institutions should support individualized strategies that are continually refined during their training. Here, drawing from our discussion as part of the 2019–2020 eLife Community Ambassador program and our experiences as ECRs, we examine the strategies individuals can adapt to strike a healthier balance between the demands of personal life and a career in research.

While many of the challenges junior academics face are systemic problems and will take a while to fix, some level of individual adjustment and planning may help ECRs more immediately and on an individual level. The rules presented here seek to empower ECRs to take action in improving their own well-being, while also providing a call to action for institutions to increase mechanisms of support for their trainees so they can thrive and move forward in their careers.

### Rule 1: Long hours do not equal productive hours

One common reason for work–life imbalance is the feeling of lagging behind as a result of the present-day competitive nature of academia. This has led to incorrectly normalized practice of overwork, due to a sense of pressure from colleagues or ourselves, contributing to increasing mental health problems in academia [3,7,9]. On the other hand, keeping a balance sets one for higher productivity and creativity [41] and long-term satisfaction with work [17,18]. It is important to focus on the benefits of work–life balance on overall well-being and to accept that performing research and building a career in academia is a long process. Taking time off should not be associated with a feeling of guilt for not working at that moment. On the contrary, it should be seen as a necessity to have good health, energy, and motivation for the next return to work. A break can result in a boost to your productivity (rate of output) [42]. Studies show output of working hours to not increase linearly after a threshold and absence of a rest day to decrease output, as long hours result in errors and accidents, as well as fatigue, stress, and sickness [43,44]. It can be challenging to cut down on work hours when you feel that there is so much to get done. We also acknowledge that there are times when putting in long hours may be needed, for example, to meet a deadline; however, keeping this behavior constant might have more disadvantages than advantages in the long term.

Having flexibility in when and where you work can help you manage tasks and feel more balanced. It is important to discuss your needs with people at work and at home, in order to establish expectations and fit your lifestyle.

### Rule 2: Examine your options for flexible work practices

Examine your relationship with your work, and try alternative schedules. Review your expected obligations, employer work hour rules, and offered benefits. Where possible, make use of modernization of work tools (such as remote work methods using digital technologies); working time is no longer exclusively based on in-person presence at the workplace, but rather the accomplishment of tasks [45,46]. The virtual office aspect can offer extensive flexibility in terms of time and location of work, reduce time spent traveling and commuting, and allow easier management of schedules and lives. Attending conferences online and giving invited talks, seminars, and interviews virtually can reduce fatigue and increase the time available for activities essential for your well-being [47,48]. Working remotely may not work for all or on many days of a week, but an overall reduction in travel is possible. In some instances, it may be difficult to know beforehand how much time you will be allocating to particular tasks in your new job, also some tasks such as fieldwork or labwork cannot be done remotely. Factor in workplace flexibility policies when looking at employment options and negotiating contracts. At the interview stage, ask your employer and prospective supervisor about flexible hours, options such as compressed workweeks, job sharing, telecommuting, or other scheduling flexibility to work in a way that best fits your efficiency and productivity. The more control you have over where and when you work, the less stressed you are likely to be. Once you know the options available to you, agree on a schedule based on your expectations and needs. Clear agreements on how and when to work are necessary to avoid conflict between work and nonwork obligations [45], so it is important to effectively communicate agreements with your managers, mentors [31], supervisors, colleagues, and also with your family. Having said this, in reality, ECRs may not always be able to negotiate salaries and benefits as conditions might be predetermined by an institution, a fellowship, or a PI’s strict expectations. Weigh the pros and cons of nonnegotiable job offers carefully. Remember that some constraints might be relaxed over time as your new employers build trust in you; therefore, continue the communication to find the best arrangements for your work.

As you try to reduce overworking and be more flexible with working arrangements, you will need to be very focused within the time frame that you have available. This is especially important as work–life balance boundaries become blurred if working from home. Setting boundaries is critical to success, as detailed below.

### Rule 3: Set boundaries to establish your workplace and time

Setting spatial and temporal boundaries around your work is important for focusing on the task in hand and preventing work from taking over other parts of your life. When you are in the office and need to focus, make sure you can work in a quiet place where colleagues are unlikely to distract you. If you work in a shared office space, communicate with those around you to let them know your needs, or if you need complete silence, then consider working in a designated space for focused work. While working from home, some may struggle to disconnect from work, step away from screens, and set clear boundaries between digital and physical settings. Screen time needs to be managed so that remote workers do not blur the lines of work and life, as that can result in discouragement and burnout. Ask your coworkers to not demand your attention toward work after a certain time in the evening. Turn off email notifications outside of working hours. By setting boundaries, you will also set an example for your coworkers and mentees. When working at home, separating your workspaces from relaxation spaces can be helpful. This way, less clutter can decrease your stress levels, and a separated space can help you to draw a line between work and family. Even carving an area on a table dedicated to your work time can help with calm and work–life balance.

In order for your resulting work to be of high quality, diligence is key. In addition to being focused on your task, you should also establish a routine and prioritize your tasks, being able to then gain more control over your time. Learning to say no is also critical. Below we expand on these issues in the context of efficiency and productivity.

### Rule 4: Commit to strategies that increase your efficiency and productivity

Many people use to-do lists and outline daily/weekly tasks, defining both work- and nonwork-related obligations that need to be accomplished. For nonwork responsibilities, devise a strategy with your family or those you live with to delegate tasks. Make sure responsibilities at home are clearly outlined and evenly distributed.

- **Manage your time.** Learning how to effectively manage your time and focus while at work is critical. Set a schedule to help in managing time, and do not forget to include buffer times between your plans, such as a coffee break with colleagues and a walk away from the bench or computer screen, to socialize and rest. Outside of busy periods, try to keep routines of work hours. Try time blocking, for example, check email and other social media (e.g., Slack) messages at specific times of the workday, and, if possible, arrange meetings at concentrated times during the day. This will maximize the amount of deep work that can be done during work hours. Sometimes, multitasking, for instance, running a few experiments at the same time or trying to work in between several meetings, may not result in great outcomes; have realistic plans and monotask if you find it better.
- **Minimize decision fatigue.** Decision fatigue refers to the deteriorating quality of decisions made by an individual after a long session of decision-making. Decision fatigue depletes self-control, which results in emotional stress, underachievement, lack of persistence, and even failures of task performance [49]. To reduce this, make the most important decisions first in your workday, and limit and simplify your choices.
- **Collaborate.** Workplace and home collaborations can take some of the load off and help in managing stress. Adjusting to teamwork or training a student may seem like extra commitments at the beginning, but, in the long run, they can help delegate some of the tasks on your calendar and help maintain a better work–life balance.
- **Do not overcommit.** Learn to say “no” [46]. Consider that accepting extra, low-impact tasks will sacrifice your nonwork time and may also take attention off your other important work appointments. Try to drop activities that drain your energy, such as nonessential meetings that do not enhance your life or career, and be efficient within this limited time with set goals.
- **Discover your own strategies.** Try to figure out what strategies work for you, and apply these to your life. Individuals respond differently to time of the day, physical conditions, and stress. Productivity may come with creative arrangements, and a high degree of organization may not work for everyone. Sometimes, improvisation and flexible schedules might be what you need.

As you begin to make decisions about the best way to manage your time, being strategic is key to prioritizing. You should aim to review your strategy and ability to stick to it often.

### Rule 5: Have a long-term strategy to help with prioritization, and review it regularly

**Fig 1:**
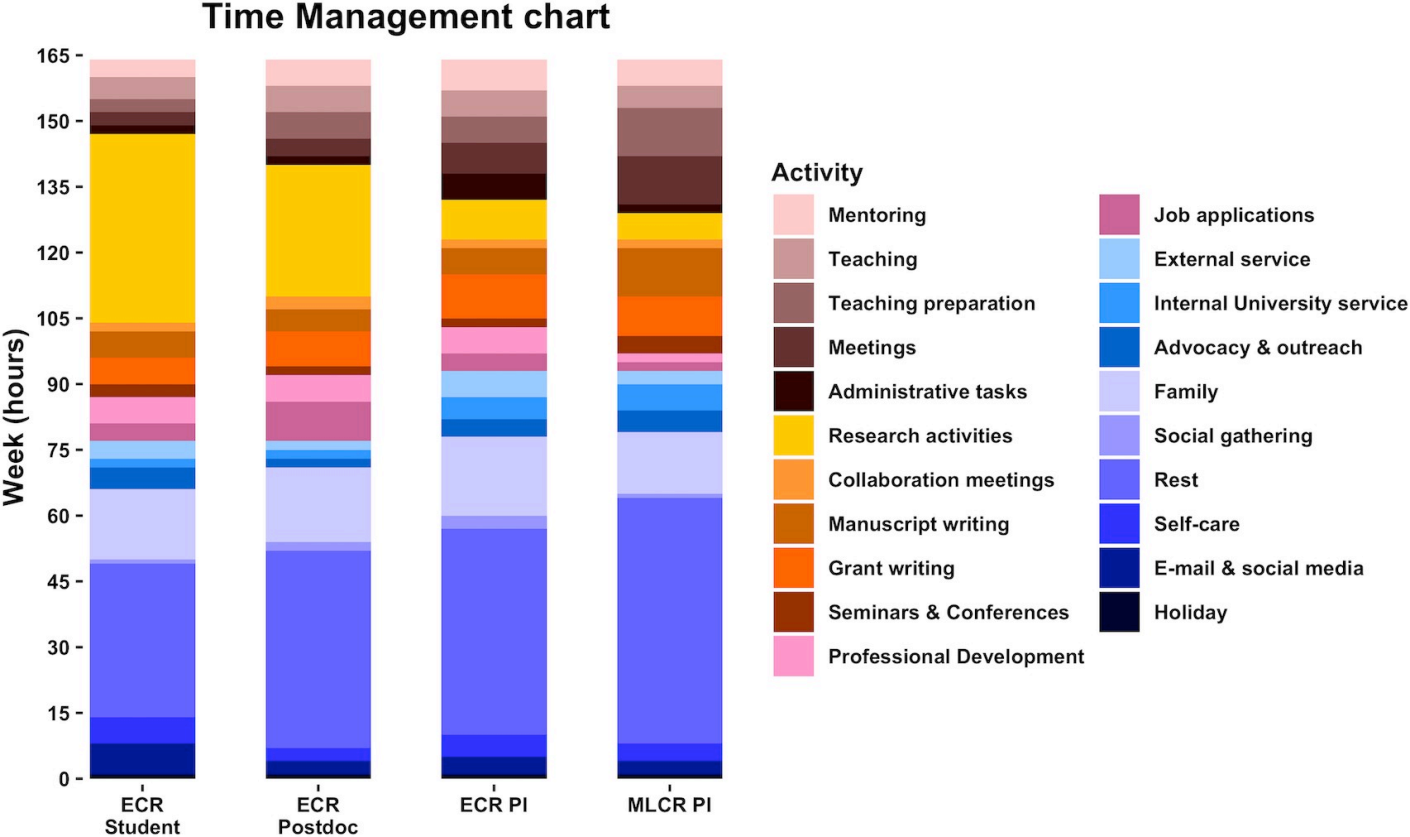
Time management charts to help monitor workload and prioritize activities. Dynamic, prospective, or retrospective weekly or monthly time management assessment charts can help researchers with improving their work–life balance by determining exactly how they spend their time. There are 164 hours in a week. Example hour allocation is shown here for academics across career stages [50]. Hours allocated will vary depending on the researcher’s disciplines (for instance, humanities versus life sciences or engineering) and circumstances such as end or beginning of semester, when approaching a deadline, or when a committee is busiest. Teaching responsibilities include course instruction and administration, including grading and evaluation. Family time includes interacting, dining, and performing housekeeping chores with family members. Research activities include performing research and literature review time. External service may include manuscript or grant reviewing and editorial tasks. Meetings may include lab/group meetings, departmental faculty meetings, or other council meetings. Self-care activities may include attending to one’s hobbies. Internal service includes department and university service. Weekends and public holidays are included in the weeks. Other tasks not included in this chart may be professional development, writing letters of recommendation, advising undergraduate students, faculty and student hiring/recruitment, marketing/public relations, fundraising, phone calls, reception/dinner, commute/travel, scheduling/planning, and reporting. ECR, early career researcher; MLCR, mid- to later career researcher; PI, principal investigator. Figure created using ggplot library in R [51].

Having a long-term strategy that considers what you want to achieve and the timelines needed to get there can help with prioritization and deciding what to take on and what to say no to. This not only includes goals linked to your research career but also what is important to you outside of work, whatever this may be. When managing your work and nonwork tasks, see how well they align with your short- and long-term goals when you are deciding on the time and energy you need to allocate to attain them. With daily tasks, starting each day with the most important task, allocating the most productive hours to important tasks, as well as grouping similar tasks might help increase productivity and efficiency. A long-term look can help justify time spent on particular tasks, such as learning new skills, which might be taking extra time now but would help reduce stress in the long term. It is important to review your strategic goals and how well you are doing regularly, updating your strategy as needed. Consider using weekly time management charts to assess your task delegation retrospectively (Fig 1). Have you been able to reach the goals you set? Did your time get taken up by other tasks? Did you use additional time to meet work goals at the expense of priorities outside of work? Are the goals you have set realistic and achievable, or do you need to make adjustments? If this appears overwhelming remember that your plans do not necessarily need to be detailed, simply keeping track of the hours spent working can be useful [26]. It is normal for priorities to change over time. Choose mentors that can help you achieve your short- and long-term goals, and consult with them regularly on your work–life balance strategies [31].

In order to do your best in life and work, you need to put yourself first. You can do that by paying attention to your eating and sleeping schedule and engaging in activities that will keep you physically healthy and stimulate your mind.

### Rule 6: Make your health a priority

You are not only defined by your work. Spending time on self-care and relaxation is a necessity in life to maintain a healthy body and mind, leading to a fulfilling lifestyle. This, in turn, will enable you to achieve peak performance and productivity in the workspace.

- **Eat a healthy diet.** A balanced diet with emphasis on fresh fruits, vegetables, and lean protein enhances the ability to retain knowledge as well as stamina and well-being. An option could be keeping fruit baskets in your office with your colleagues.
- **Get enough sleep.** Lack of sleep increases stress, and associated fatigue is linked to poor work–life balance [52]. One potential way to improve sleep quality is to avoid using personal electronic devices, such as smartphones and tablets, during your personal and other nonwork times, particularly right before going to sleep as screen time is associated with less and poorer quality rest [53,54].
- **Prioritize your physical and mental health.** Set time aside for individual or group physical activities of your choice. Schedule specific times for social activities and exercise to unwind, by arranging ahead of time with others or signing up to regular classes, making the plans harder to cancel. Using the gym at your workplace during a break can freshen you. Or you can bike or jog to work if safe to have some daily exercise. Equally important is dedicated time for your mental health. Reading a book, listening to music, gardening, many other activities, or if you prefer, regularly talking to a therapist could help you disengage from work, enjoy other aspects of life, rest, process, and recharge.
- **Try meditation or mindfulness exercises.** Meditation can reduce stress and increase productivity [29]; it will help you focus your thoughts and develop more self-awareness. If you are aware of when and why you are stressed or exhausted, these feelings become a trigger for you to lean into a boundary such as taking a screen break, going for a walk, or simply resting your eyes for 15 minutes before jumping back into a task or meeting. You can do meditation or yoga at home for short intervals. Do what is realistic for your life at the time and what helps you along.
- **Make time for your hobbies and relaxation.** Set aside time each day for an activity that you enjoy [28,55,56]. Discover activities you can do with your partner, family, or friends—such as hiking, dancing, or taking cooking classes. Listen to your favorite music at work to foster concentration, reduce stress and anxiety, and stimulate creativity [57].

While your work is important, you will be much happier if you schedule some social time into your week. This is a simple need, and methods vary from person to person, but the common goal is to increase your sense of connection and belonging, satisfaction with life, and/or energy.

### Rule 7: Regularly interact with family and friends

Your work schedule does not need to lead to loss of your personal relationships. Scheduling time off to meet in-person or interact online with your loved ones in advance will make it harder to cancel plans in favor of working longer. As an example of good practice, most parents, even in academia, need to schedule their time around family responsibilities, which actually obliges them to maintain a work–life balance; they typically do not overstay at work every day, take the weekends off, and use annual leave. Meeting with friends and family will provide a chance to reconnect with them and your shared values. If you live in a country different from your family and friends, it is important to keep in touch using online audiovisual call and chat technologies. Other ways to relax include taking walks with loved ones, being out in nature, or playing board games. Social downtime can help replenish a person’s attention and motivation, encourages productivity and creativity, and is essential to both achieve our highest levels of performance and form stable memories in everyday life [58].

In addition to spending time for yourself and with family and friends, engaging in activities that are important to you, even when these activities are demanding, can bring a needed sense of achievement and satisfaction.

### Rule 8: Make time for volunteer work or similar commitments that are important and meaningful to you

Many find additional engagements outside of their day to day jobs both important and rewarding. These activities would not be considered hobbies or relaxation, examples may include volunteering for the local community (e.g., at pet shelters, food banks, and environmental efforts), regional and online communities (e.g., student advocacy groups), time on boards or committees outside of work (e.g., acting as treasurer or secretary of a club), and learning a new language when you have moved to a new place. Many ECRs enjoy taking their work one step forward to volunteer with organizations focused on the societal value or impact of their work. This can help expand your perspective as an ECR working on a particular research topic, by understanding the broader picture of what you are working on and why and giving it a human impact dimension. Others may opt to volunteer in activities that are entirely independent of their research, which can provide opportunities to clear your mind for a good period of time and boost your mood. Although these activities add extra work to your schedule, if they are important to you, then you might find it difficult to find balance without the sense of achievement and reward they bring. However, when under pressure from work and home, finding time for these activities can be challenging—remember that work–life balance needs to be continually reassessed; consider taking a break if you need to and revisiting these extra commitments at a better time.

In addition to advisors and departments, institutions can take measures to support ECRs and provide them with necessary resources to thrive. They should also create a culture where asking for help is encouraged, and support for the well-being of researchers exists at their institution.

### Rule 9: Seek out or help create peer and institutional support systems

Support systems are also critical to your success, and building more than one will increase your chances of success and balance overall [59]. At work, join forces with coworkers who can cover for you—and vice versa—when family conflicts arise. At home, enlist trusted friends and loved ones to pitch in with childcare or household responsibilities when you need to work overtime or travel. Seek support in academic communities and organizations who are working on mental health and well-being. For instance, PhD balance is a community space for academics to learn from shared experiences, to openly discuss and receive help for difficult situations, and to create resources and connect with others [60]. Dragonfly Mental Health, a nonprofit organization, strives to improve mental health care access and address the unhealthy culture pervading academia [61]. Everyone may need help from time to time. If life feels too chaotic to manage and you feel overwhelmed, talk with a professional, such as a counselor or other mental health provider. If your employer offers an employee assistance program, take advantage of available services. Joining a support and peer mentorship group, such as graduate, postdoctoral or faculty Slack communities [31], or working parents seeking and sharing work–life balance strategies, provides at least two key advantages: an opportunity to vent to people who truly understand your experiences and the ability to strategize with a group about how to improve your situation. A combination of these steps will help researchers to improve their work–life balance.

Finally, if your ability to effectively implement the advice in Rules 1 to 8 is constrained by the culture in your lab or pressure from the academic system, seek support from mentors, and advocate for yourself and for the change you would like to see.

### Rule 10: Open a dialogue about the importance of work–life balance and advocate for systemic change

Spreading awareness and promoting good practice for managing work–life balance are essential toward shifting the prevailing culture away from current excellence at any cost practices. While major change is only likely to come about with a coordinated shift in the way that research laboratories, institutions, publishers, funders, and governments assess research endeavors at a broadscale, there is much that can be done at smaller scales to improve the culture at institutions and within labs [62]. Leverage the support of communities that empower ECRs to participate in advocating for the importance of mental well-being in academia through research and programs (see Rule 9). Discussions on work–life balance can also be initiated through seminars and courses. You can ask for, or if you plan to get more involved, organize workshops and training in your institute for ECRs. Another way to encourage collective work–life balance could be to host activities such as family and employee sports, outdoor movies, or picnic events encouraging family-friendly time and team building. Advocate for policies in your workplace that can help reduce conflict between work and other responsibilities, for example, childcare services or pet-friendly workspaces. To advocate at larger scales, you can join graduate/postdoctoral researcher associations, unions, or work councils to actively pursue work–life balance–friendly policies and employment contracts at institutes and through funding agencies. For instance, institutions and funding agencies that do not encourage the traditional gender roles allowing both men and women to take family leave, see better work–life balance, and reduced work–life conflict [63,64]. If the culture in your research lab constrains your ability to manage your work–life balance in a way you find satisfactory, shifting departmental and institutional attitudes and policies can put pressure on PIs to build a more supportive work culture via steps outlined elsewhere [11,12,31–33]. Although organizational culture cannot be changed overnight, changes in policy can go a long way in creating a culture that aids work–life balance in the academic workplace [62–64].

## Conclusions

Most academic jobs come with flexible working hours, which can be advantageous when researchers attempt to balance the competing obligations in their lives. Yet, ECRs typically work significantly longer than the normal working hours of academic employment contracts [65]. How researchers spend their time has major impacts on their well-being, productivity, and professional scale of impact and those of their mentees, family, colleagues, and institutions in the short and long term. Academic culture has normalized and ignored overworking often at the expense of a social life, or of even greater concern, at the expense of researchers’ health and well-being. It is important for all academic researchers, institutions, and funding agencies to credit service and administrative activities, to acknowledge difficulties in satisfying work- and nonwork-related obligations in academic careers, and support diverse strategies to attain work–life balance [29,30]. It is imperative to examine work–life balance practices by ECRs, suggest improvements, and integrate these into employment and promotion offers. Here, we provided recommendations for ECRs to improve management of the balance between their professional and personal lives, but striking a healthy work–life balance is not a one-shot deal. Managing work–life balance is a continuous process as your family, interests, and work life change. Working long hours does not equate to working better. Regularly examine your priorities—and, if necessary, make changes—to ensure you stay on track. Ultimately, for the benefit of researchers and the important work that they do, both individuals and institutions need to make health and well-being a priority.

## References

[1] Kinman G. Doing More with Less? Work and Wellbeing in Academics. Somatechnics. 2014;4:219–35. doi: 10.3366/soma.2014.0129

[2] Powell K. Young, talented and fed-up. Nature. 2016;538:446–9. doi:doi: 10.1038/538446a 27786221

[3] Trust Wellcome. What researchers think about the culture they work in. 2020 pp. 1–50. Available from: https://wellcome.ac.uk/reports/what-researchers-think-about-research-culture

[4] Herbert DL, Coveney J, Clarke P, Graves N, Barnett AG. The impact of funding deadlines on personal workloads, stress and family relationships: a qualitative study of Australian researchers. BMJ Open. 2014;4:e004462. doi: 10.1136/bmjopen-2013-004462 24682577 PMC3975760

[5] Fernandes JD, Sarabipour S, Smith CT, Niemi NM, Jadavji NM, Kozik AJ, et al. Research Culture: A survey-based analysis of the academic job market. Elife. 2020;9:e54097. 10.7554/eLife.54097 32530420 PMC7360372

[6] Editorial. The mental health of PhD researchers demands urgent attention. Nature. 2019;575:257–8. doi: 10.1038/d41586-019-03489-1 31723298

[7] Bleasdale B. Researchers pay the cost of research. Nat Mater. 2019;18:772–2. doi: 10.1038/s41563-019-0443-z 31332321

[8] Woolston C. Postdoc survey reveals disenchantment with working life. Nature. 2020;587:505–8. doi: 10.1038/d41586-020-03191-7 33208965

[9] Woolston C. Postdocs under pressure: ‘Can I even do this any more?’. Nature. 2020;587:689–92. doi: 10.1038/d41586-020-03235-y 33230311

[10] Woolston C. ‘The problem is greater than it’s ever been’: US universities urged to invest in mental-health resources. Nature. 2021;590:171–2. doi: 10.1038/d41586-021-00229-2 33495614

[11] Maestre FT. Ten simple rules towards healthier research labs. PLoS Comput Biol. 2019;15:e1006914. doi: 10.1371/journal.pcbi.1006914 30973866 PMC6459491

[12] Maestre FT. Seven steps towards health and happiness in the lab. Nature. 2018. doi: 10.1038/d41586-018-07514-7

[13] Casper WJ, Vaziri H, Wayne JH, DeHauw S, Greenhaus J. The jingle-jangle of work–nonwork balance: A comprehensive and meta-analytic review of its meaning and measurement. J Appl Psychol. 2018;103:182–214. doi: 10.1037/apl0000259 29016161

[14] Heijstra TM, Rafnsdottir GL. The Internet and academics’ workload and work–family balance. Internet High Educ. 2010;13:158–63. doi: 10.1016/j.iheduc.2010.03.004

[15] Currie J, Eveline J. E-technology and work/life balance for academics with young children. High Educ. 2011;62:533–50. doi: 10.1007/s10734-010-9404-9

[16] The Whole Scientist: The Jackson Laboratory. Available from: https://www.jax.org/education-and-learning/education-calendar/2020/11-november/the-whole-scientist-bh

[17] Aarnikoivu M, Nokkala T, Siekkinen T, Kuoppala K, Pekkola E. Working outside academia? Perceptions of early-career, fixed-term researchers on changing careers. Eur J High Educ. 2019;9:172–89. doi: 10.1080/21568235.2018.1548941

[18] Dorenkamp I, Weiß E-E. What makes them leave? A path model of postdocs’ intentions to leave academia. High Educ. 2018;75:747–67. doi: 10.1007/s10734-017-0164-7

[19] Balven R, Fenters V, Siegel DS, Waldman D. Academic entrepreneurship: the roles of identity, motivation, championing, education, work-life balance, and organizational justice. Acad Manag Perspect. 2018;32:21–42. doi: 10.5465/amp.2016.0127

[20] Bothwell E. Work-life balance survey 2018: long hours take their toll on academics. In: Times Higher Education [Internet]. [cited 2020 Jul 9]. Available from: https://www.timeshighereducation.com/features/work-life-balance-survey-2018-long-hours-take-their-toll-academics

[21] Johnson S, Willis S, Evans J. An examination of stressors, strain and resilience in academic and non-academic UK university job roles. Int J Stress Manag. 2019;26:162–72.

[22] Evans TM, Bira L, Beltran Gastelum J, Weiss LT, Vanderford NL. Evidence for a mental health crisis in graduate education. Nat Biotechnol. 2018;36:282–4. doi: 10.1038/nbt.4089 29509732

[23] Loissel E, Deathridge J, King SRF, Pérez Valle H, Rodgers PA. Mental Health in Academia. In: collection by eLife [Internet]. eLife. Sciences Publications Limited; 23 Oct 2019 [cited 2020 Jul 21]. Available from: https://elifesciences.org/collections/ad8125f3/mental-health-in-academia doi: 10.7554/eLife.52881

[24] Woolston C. Full-time is full enough. Nature. 2017;546:175–7.

[25] Woolston C. A love–hurt relationship. Nature. 2017;550:549–52. doi: 10.1016/j.joca.2017.03.011 28336452 PMC5518889

[26] Foltz A. Why clocking my work hours shifted my work-life balance. Science. 2020 [cited 2021 Mar 1]. 10.1126/science.caredit.abc5428

[27] Lechler R. Diversity, creativity, and flexibility will be needed from the next generation of medical scientists. Lancet. 2017;389. doi: 10.1016/S0140-6736(17)31225-4 28236832

[28] Pineda A. Building a meditation routine for a more productive, creative and happier scientific life. Nature. 2020 [cited 2021 Mar 1]. doi: 10.1038/d41586-020-02537-5 32884144

[29] Cannizzo F, Mauri C, Osbaldiston N. Moral barriers between work/life balance policy and practice in academia. J Cult Econ. 2019;12:251–64. doi: 10.1080/17530350.2019.1605400

[30] Owens J, Kottwitz C, Tiedt J, Ramirez J. Strategies to attain faculty work-life balance. Build Healthy Acad Communities J. 2018;2:58–73. doi: 10.18061/bhac.v2i2.6544

[31] Sarabipour S, Hainer SJ, Nur Arslan F, De Winde CM, Furlong E, Bielczyk N, et al. Building and sustaining mentor interactions as a mentee. FEBS J. 2021 [cited 2021 May 2]. doi: 10.1111/febs.15823 33818917 PMC8490489

[32] Loissel E. Mental Health in Academia: Shedding light on those who provide support. eLife. 2020;9:e64739. doi: 10.7554/eLife.64739 33226339 PMC7682984

[33] Davla S. How to build a healthy student-supervisor relationship in graduate school. In: Prelights [Internet]. 1 Apr 2020 [cited 2020 Sep 2]. Available from: https://prelights.biologists.com/highlights/supervising-the-phd-identifying-common-mismatches-in-expectations-between-candidate-and-supervisor-to-improve-research-training-outcomes/

[34] Lewis RA. Work-life balance in academia: Experiences of lecturers in Switzerland. Int J Bus Manag. 2016;4:69–84. 10.20472/BM.2016.4.1.004

[35] Dorenkamp I, Süß S. Work-life conflict among young academics: antecedents and gender effects. Eur J High Educ. 2017;7:402–23. doi: 10.1080/21568235.2017.1304824

[36] Denson N, Szelényi K, Bresonis K. Correlates of work-life balance for faculty across racial/ethnic groups. Res High Educ. 2018;59:226–47. doi: 10.1007/s11162-017-9464-0

[37] Hogan V, Hogan M, Hodgins M, Kinman G, Bunting B. An examination of gender differences in the impact of individual and organisational factors on work hours, work-life conflict and psychological strain in academics. Ir J Psychol. 2014;35:133–50. doi: 10.1080/03033910.2015.1011193

[38] Bozzon R, Murgia A, Poggio B, Rapetti E. Work–life interferences in the early stages of academic careers: The case of precarious researchers in Italy. Eur Educ Res J. 2017;16:332–51. doi: 10.1177/1474904116669364

[39] O’laughlin EM, Bischoff LG. Balancing parenthood and academia: work/family stress as influenced by gender and tenure status. J Fam Issues. 2005;26:79–106. doi: 10.1177/0192513X04265942

[40] Allen TD, Herst DEL, Bruck CS, Sutton M. Consequences associated with work-to-family conflict: A review and agenda for future research. J Occup Health Psychol. 2000;5:278–308. doi: 10.1037//1076-8998.5.2.278 10784291

[41] James A. Work-Life ‘Balance’, Recession and the Gendered Limits to Learning and Innovation (Or, Why It Pays Employers To Care). Gend Work Organ. 2014;21:273–94. doi: 10.1111/gwao.12037

[42] Oyama Y, Manalo E, Nakatani Y. The Hemingway effect: How failing to finish a task can have a positive effect on motivation. Think Skills Creat. 2018;30:7–18. doi: 10.1016/j.tsc.2018.01.001

[43] Pencavel J. The Productivity of Working Hours. Econ J. 2015;125:2052–76. doi: 10.1111/ecoj.12166

[44] Collewet M, Sauermann J. Working hours and productivity. Labour Econ. 2017;47:96–106. doi: 10.1016/j.labeco.2017.03.006

[45] Burroughs Wellcome Fund, Howard Hughes Medical Institute. Making the right moves: a practical guide to scientifıc management for postdocs and new faculty. 2nd ed. 2019. Available from: https://www.hhmi.org/sites/default/files/Educational%20Materials/Lab%20Management/Making%20the%20Right%20Moves/moves2.pdf

[46] Hinton AO Jr, McReynolds MR, Martinez D, Shuler HD, Termini CM. The power of saying no. EMBO Rep. 2020;21:e50918. doi: 10.15252/embr.202050918 32596868 PMC7332800

[47] Lirio P. Taming travel for work-life balance in global careers. J Glob Mobil. 2014;2:160–82. 10.1108/JGM-06-2013-0028

[48] Sarabipour S. Research Culture: Virtual conferences raise standards for accessibility and interactions. eLife. 2020;9:e62668. doi: 10.7554/eLife.62668 33143847 PMC7641586

[49] Reiner BI, Krupinski E. The Insidious Problem of Fatigue in Medical Imaging Practice. J Digit Imaging. 2012;25:3–6. doi: 10.1007/s10278-011-9436-4 22143410 PMC3264708

[50] Ziker J. The Long, Lonely Job of Homo Academicus. In: Boise State University [Internet]. 31 Mar 2014 [cited 2020 Oct 18]. Available from: https://www.boisestate.edu/bluereview/faculty-time-allocation/

[51] Wickham H. ggplot2: elegant graphics for data analysis. 2016. Available from: https://books.google.com/books?hl=en&lr=&id=XgFkDAAAQBAJ&oi=fnd&pg=PR8&ots=spY07U8X3P&sig=Vw5aHFonM3Ee56OTEuWCfgUXA-c#v=onepage&q&f=false

[52] Gander P, Briar C, Garden A, Purnell H, Woodward A. A Gender-Based Analysis of Work Patterns, Fatigue, and Work/Life Balance Among Physicians in Postgraduate Training. Acad Med. 2010;85:1526–36. doi: 10.1097/ACM.0b013e3181eabd06 20736682

[53] Wu X, Tao S, Zhang Y, Zhang S, Tao F. Low Physical Activity and High Screen Time Can Increase the Risks of Mental Health Problems and Poor Sleep Quality among Chinese College Students. PLoS ONE. 2015;10:e0119607. doi: 10.1371/journal.pone.0119607 25786030 PMC4364939

[54] Christensen MA, Bettencourt L, Kaye L, Moturu ST, Nguyen KT, Olgin JE, et al. Direct Measurements of Smartphone Screen-Time: Relationships with Demographics and Sleep. PLoS ONE. 2016;e0165331:11. doi: 10.1371/journal.pone.0165331 27829040 PMC5102460

[55] Powell K. Work–life balance: Break or burn out. Nature. 2017;545:375–7. doi: 10.1038/nj7654-375a

[56] Woolston C. Leisure activities: The power of a pastime. Nature. 2015;523:117–9. doi: 10.1038/nj7558-117a 26151070

[57] Rosen J. How a hobby can boost researchers’ productivity and creativity. Nature. 2018;558:475–7. doi: 10.1038/d41586-018-05449-7 29915421

[58] Jabr F. Why Your Brain Needs More Downtime. In: Scientific American [Internet]. 15 Oct 2013 [cited 2020 Jul 8]. Available from: https://www.scientificamerican.com/article/mental-downtime/

[59] Supporting the Whole Student: Mental Health and Well-Being in STEMM Undergraduate and Graduate Education. In: The National Academy of Sciences, Engineering & Medicine [Internet]. 2020 [cited 2020 Jul 6]. Available from: https://www8.nationalacademies.org/pa/projectview.aspx?key=51350

[60] PhD Balance: A community empowering graduate students. 2020 [cited 2020 May 29]. Available from: https://www.phdbalance.com/

[61] Dragonfly Mental Health. [cited 2020 May 29]. Available from: http://dragonflymentalhealth.com/about-us/

[62] Casci T, Adams E. Setting the right tone. eLife. 2020;9:e55543. doi: 10.7554/eLife.55543 32036857 PMC7010405

[63] Feeney MK, Stritch JM. Family-Friendly Policies, Gender, and Work–Life Balance in the Public Sector. Rev Public Pers Adm. 2017 [cited 2021 Mar 5]. doi: 10.1177/0734371X17698189 29046599 PMC5633037

[64] Skinner N, Chapman J. Work-life balance and family friendly policies. Evid Base. 2013;2017:1–17. 10.4225/50/558217B4DE473

[65] Woolston C. PhDs: the tortuous truth. Nature. 2019;575:403–6. doi: 10.1038/d41586-019-03459-7 31723297

